# A Recent Review on Cancer Nanomedicine

**DOI:** 10.3390/cancers15082256

**Published:** 2023-04-12

**Authors:** Paras Mani Giri, Anurag Banerjee, Buddhadev Layek

**Affiliations:** Department of Pharmaceutical Sciences, School of Pharmacy, College of Health Professions, North Dakota State University, Fargo, ND 58105, USA

**Keywords:** cancer, chemotherapy, inorganic nanoparticles, liposomes, nanoparticles, targeted drug delivery, theranostics

## Abstract

**Simple Summary:**

The advancement of nanotechnology over the last three decades has given new hope to cancer management. The first FDA-approved nanomedicine (DOXIL) was made available in the market in 1995. Since then, numerous nanocarriers have been synthesized and extensively evaluated for antitumor efficacy to establish them as premier therapeutic tools. Even though nanomedicine is one of the most promising breakthroughs in the modern era of medicine, several challenges are still faced during scaling up from a laboratory setup to a clinical arrangement. In this review, we describe and compare various types of nanoparticles and their role in advancing cancer treatment. Moreover, we highlight various nanomedicines currently available for cancer therapy and nanoformulations that are through various stages of clinical testing.

**Abstract:**

Cancer is one of the most prevalent diseases globally and is the second major cause of death in the United States. Despite the continuous efforts to understand tumor mechanisms and various approaches taken for treatment over decades, no significant improvements have been observed in cancer therapy. Lack of tumor specificity, dose-related toxicity, low bioavailability, and lack of stability of chemotherapeutics are major hindrances to cancer treatment. Nanomedicine has drawn the attention of many researchers due to its potential for tumor-specific delivery while minimizing unwanted side effects. The application of these nanoparticles is not limited to just therapeutic uses; some of them have shown to have extremely promising diagnostic potential. In this review, we describe and compare various types of nanoparticles and their role in advancing cancer treatment. We further highlight various nanoformulations currently approved for cancer therapy as well as under different phases of clinical trials. Finally, we discuss the prospect of nanomedicine in cancer management.

## 1. Introduction

Cancer is the second biggest cause of mortality, accounting for one in every six fatalities. In 2020, approximately there were 19.3 million new cases of cancer worldwide (18.1 million excluding nonmelanoma skin cancer) and over 10 million cancer deaths (9.9 million excluding nonmelanoma skin cancer); 70% were in low- and middle-income countries [1]. The poor prognosis of cancer is primarily attributed to the absence of early diagnostic tools and the lack of effective therapy. Current cancer diagnostic and treatment paradigms involve standardized screening methods for a restricted number of cancer types, followed by treatment consisting of surgical intervention, radiation, and chemotherapy [2]. In addition to these traditional treatments, immunotherapy, hormone therapy, gene therapy, and stem cell therapy have been widely studied in recent years. 

Among all these available strategies, chemotherapy is still the most effective and economical option, particularly for advanced diseases. Despite considerable biological and clinical improvements in recent years that have enhanced treatment outcomes in cancer patients, most contemporary chemotherapeutic agents have unwanted and severe side effects that can affect the continuation of treatment and negatively impact a patient’s quality of life [3]. Chemotherapeutics primarily act by inhibiting various stages of mitosis, allowing them to target rapidly dividing cancer cells. Since traditional chemotherapeutics lack adequate cancer cell specificity, a high dose of these medications must be provided to obtain therapeutic levels, resulting in various dose-dependent side effects since other fast-dividing healthy cells are also impacted. Some notable side effects of chemotherapeutics include neuropathy, nausea, general discomfort, myelosuppression, alopecia, nephrotoxicity, and cardiotoxicity. Moreover, most chemotherapeutic agents lack desired aqueous solubility, resulting in formulation challenges and unfavorable pharmacokinetic profiles, including poor bioavailability [4]. Another significant issue is that cancer cells often develop resistance to chemotherapy treatments. 

Nanotechnology has been shown to circumvent many limitations of traditional chemotherapy to an extent and serves as a valuable tool for improving overall cancer treatment outcomes. Generally, drug molecules are dissolved, adsorbed, entrapped, encapsulated, or attached to nanomatrices. Due to their nanoscale dimensions (typically <500 nm) and large surface-area-to-volume ratios, nanocarriers can favorably influence the fundamental properties and biological activity of their payloads [5]. Additionally, nanoparticles can enhance the bioavailability of medication with poor aqueous solubility while offering tumor-selective accumulation of their payloads. Most importantly, nanoformulations promote preferential tumor accumulation of chemotherapeutic agents, enhancing therapeutic efficacy while reducing systemic toxicity. 

**Figure 1:**
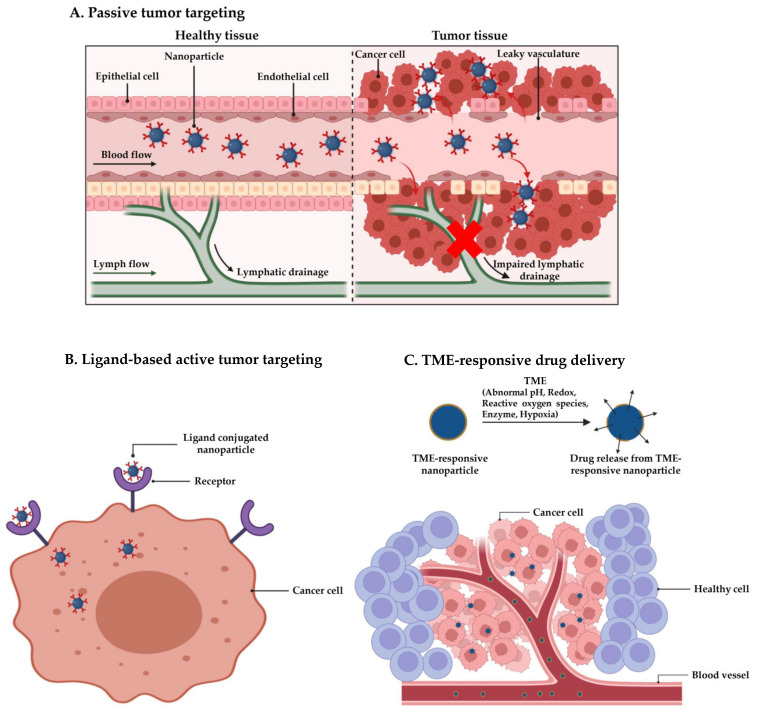
Nanocarrier-mediated tumor targeting. (**A**) Passive tumor targeting, (**B**) ligand-based active tumor targeting, and (**C**) TME-responsive drug delivery. Created with BioRender.com.

The use of nanocarriers for cancer therapy primarily relies on enhanced permeability and retention (EPR) effects first observed by Matsumura and Maeda in 1986 [6]. The EPR effects depend on high vascular permeability and reduced lymphatic outflow in solid tumors to enable passive targeting and long-term retention of nanoparticles at the tumor site (Figure 1A). Consequently, nanomedicines significantly improve therapeutic outcomes while minimizing the dose-dependent toxicity of chemotherapeutic agents. Over the years, EPR effects have become the backbone of nanocarrier-based cancer therapy and have led to the approval of several nanomedicines [7]. Many other nanocarrier-based therapies, such as ABI-009 (albumin-bound rapamycin nanoparticles) [8], CPX-351 (cytarabine and daunorubicin encapsulated liposomes) [9], and DoceAqualip (nanosomal docetaxel lipid suspension) [10], have shown promising anticancer efficacy in clinical trials.

Although passive targeting-based nanomedicines have shown great enthusiasm in cancer therapy, the major disadvantages of EPR-based formulations include their nonspecific distribution, insufficient tumor accumulation, and intra- and inter-tumoral as well as inter-individual heterogeneity. In addition, multiple stromal factors such as dense extracellular matrix, high interstitial fluid pressure, growth-induced solid stress, and hypoxia can further aggravate heterogeneity in EPR-based tumor targeting. As a result of the wide variations in tumor blood flow and vascular permeability, the EPR effect may not be applicable to all solid tumors. Clinically, tumor size-related obstruction of tumor blood flow is frequently observed; small early-stage tumors have a more consistent EPR effect, whereas large tumors exhibit greater variability in the EPR effect. A recent study also revealed that 97% of nanoparticles enter the tumor due to active transport via trans-endothelial pathways from blood vessel cells to tumor tissue [11]. 

In this context, researchers have concentrated on developing next-generation nanocarriers with enhanced pragmatism, such as ligand-based active tumor-targeting (Figure 1B) and tumor microenvironment (TME)-responsive drug delivery (Figure 1C). The ligand-based targeting strategy relies on the direct interaction of ligands on nanocarriers which selectively interact with the overexpressed receptors or antigens on cancer cells and enhance nanocarrier uptake. For instance, cetuximab conjugated nanoparticles were developed to actively target epidermal growth factor receptor (EGFR) overexpressing colon cancer cells [12]. However, the lack of such receptors in healthy cells decreases the interactions between normal cells and ligand-decorated nanocarriers, preventing uptake by normal cells. On the other hand, TME-responsive delivery systems offer on-demand drug release in response to the altered physiological features of TME, which differs from healthy tissues. For instance, a solid tumor creates a microenvironment with an acidic pH environment, hypoxic conditions, an altered redox environment, and elevated level of reactive oxygen species (ROS) [13,14].

In addition, the physicochemical characteristics of nanocarriers and their interaction with biological systems can be modulated favorably by altering their composition, shape, size, and surface characteristics. For example, the surface modification of nanoparticles with polyethylene glycol (PEG) improves the drug’s circulatory half-life, resulting in its enhanced bioavailability at the tumor site [15]. 

## 2. Types of Nanocarriers

**Figure 2:**
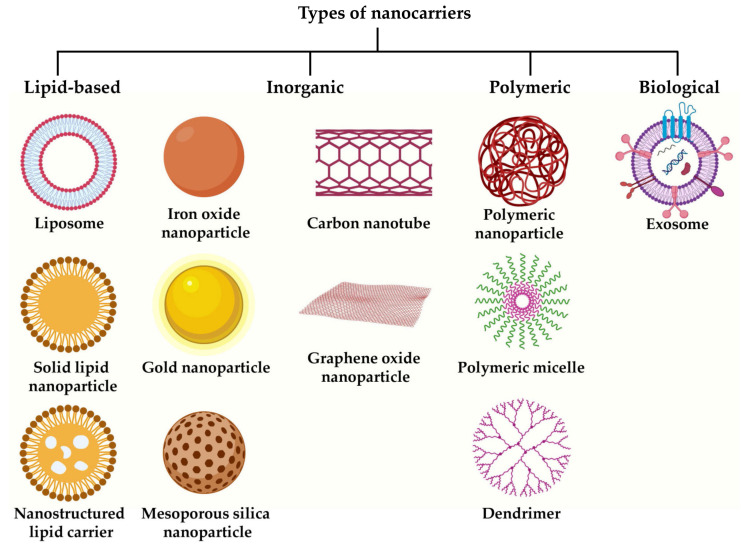
Types of nanocarriers frequently used for cancer therapy. Created with BioRender.com.

Up to now, various nanoplatforms have been explored in oncology, including lipid-based, inorganic materials, polymer-based, and even biological nanocarriers such as exosomes. Inorganic nanocarriers can be further subclassified into different categories, such as metallic nanocarriers, mesoporous silica nanocarriers, carbon nanotubes, and graphene oxide nanoparticles. The most prevalent classes of nanomedicines employed for clinical and preclinical applications are listed in Figure 2. The following section briefly discusses each of these classes with suitable examples.

### 2.1. Lipid-Based Nanocarriers

Among various nanocarriers, lipid-based formulations have been extensively investigated for cancer therapy [16,17,18]. As drug carriers, they offer myriad advantages, including excellent biocompatibility, biodegradability, superior bioavailability, flexibility to incorporate both hydrophilic and hydrophobic therapeutics with large drug-loading capacities, prolonged and controlled drug release, and a set of programable physical and chemical features to control their biological properties [8,16,19]. Based on the composition and physicochemical features, lipid-based nanocarriers are divided into three main categories: liposomes, solid lipid nanoparticles (SLNs), and nanostructured lipid carriers (NLCs). 

#### 2.1.1. Liposomes

Liposomes are lipid vesicles composed of one (unilamellar) or many (multilamellar) concentric bilayers enclosing an aqueous core. Because of their unique structure, liposomes can be loaded with hydrophilic and lipophilic molecules, enabling the administration of a wide array of drugs. Liposomes are considered safe nanocarriers since they are made of biocompatible and biodegradable lipids such as phospholipids and cholesterol [20,21]. Furthermore, they can be easily functionalized with various moieties to extend circulation half-life, enable target specificity, facilitate cellular uptake, and even provide stimuli-responsive drug release [22,23,24,25,26]. These beneficial properties of liposomes pave the way for their successful clinical implementation. Several liposomal formulations are currently in routine clinical use, and numerous others are in various stages of clinical trials or awaiting approval [16,27,28]. 

Besides the successful clinical translation of several liposomal formulations for cancer therapy, liposomes are still actively investigated as multimodal nanocarriers. Conventional liposomes minimize adverse side effects of chemotherapeutics by modulating pharmacokinetics and biodistribution profiles to improve drug delivery to tumor tissue. However, the liposomal formulations are prone to quick removal from circulation, limiting their therapeutic efficacy. The fast clearance of liposomes is attributed to the opsonization of plasma components and subsequent uptake by the reticuloendothelial system (RES), mainly in the liver and spleen [19,29]. The initial signal for liposome clearance is the binding of opsonin to the liposome surface; in fact, the RES does not detect the liposomes themselves but instead identifies opsonin linked to the liposomes’ surface. Stealth liposomes were synthesized using PEG and other hydrophilic polymers as steric stabilizers to increase liposome stability and blood circulation times. PEG coatings on liposomes protect the surface against aggregation, opsonization, and phagocytosis, thereby extending systemic circulation [30]. In a clinical study, Doxil^®^ (PEGylated doxorubicin liposomes) showed biphasic clearance with one-third of the administered dose cleared from the plasma during initial distribution half-life (1–3 h), and the remaining Doxil^®^ was eliminated slowly, with a terminal half-life of 42–46 h. However, the initial distribution half-life of free doxorubicin was approximately 5 min, and the terminal half-life was 25–30 h. Furthermore, clearance and volume of distribution of doxorubicin drastically reduced following liposome encapsulation, resulting in approximately 300-fold higher AUC than the free drug [31].

Nowadays, active targeting has become the mainstay of cancer therapy, promoting preferential drug accumulation at the tumor sites while sparing healthy tissues, leading to enhanced efficacy and minimizing unwanted toxicities. Therefore, liposomes are often functionalized with various tumor-specific ligands or antibodies, including folic acid [32], hyaluronic acid [33], antibodies [34], and aptamers [35], to achieve active tumor targeting. Liposomes and other nanocarriers can also be constructed with specific materials to facilitate chemotherapeutics release in response to TME. In a recent study, Nunes et al. [36] formulated folate-conjugated pH-responsive liposomes for tumor-specific delivery of irinotecan. This formulation exhibited a pH-dependent sustained drug release profile with better uptake by the tumor cells. Notably, the dual-functionalized liposomes showed significantly higher antitumor efficacy against colorectal cancer than the pH-sensitive system alone or the free irinotecan. 

Besides their drug delivery potential, liposome-based formulations have also been investigated for theranostic purposes. Indocyanine green, a near-infrared (NIR) dye, and doxorubicin-co-encapsulated thermoresponsive liposomes were formulated depending on natural phase-change material to develop an image-guided multimodal drug delivery platform [37]. Additional folic acid and gadolinium modifications provide liposomes with active tumor targeting and imaging capabilities. The liposomal formulation exhibited NIR-triggered drug release and trimodal (fluorescence, magnetic resonance imaging, MRI, and photoacoustic)-guided combination chemotherapy, photodynamic therapy (PDT), and photothermal therapy (PTT). The prepared liposomes demonstrated effective tumor accumulation and inhibition in the HeLa tumor xenograft mice model without visible toxicity.

#### 2.1.2. Solid Lipid Nanoparticles (SLNs)

SLNs comprise a solid lipid core coated with a surfactant layer to enhance their stability in the aqueous environment. Lipid materials used for SLN preparation include triglycerides (e.g., tristearin, tripalmitin, trimyristin, and trilaurin), partial glycerides (e.g., glyceryl behenate, glyceryl stearate, and glyceryl palmitostearate), fatty acids (e.g., stearic acid, palmitic acid, and capric acid), steroids (e.g., cholesterol), and waxes. The frequently used surfactants are poloxamers, lecithin, sodium glycocholate, polysorbates, sorbitan esters, and mixtures [16]. Although a wide range of lipids and surfactants could be used for SLN preparation, the proper selection of core lipids and surfactants is crucial, since they are known to affect the size, charge, storage stability, drug-loading, and drug-release profile of SLNs [38]. 

By fusing all the beneficial traits of polymeric nanoparticles, liposomes, and microemulsion, SLNs have altered the dimension of drug delivery. The key advantages of SLNs as a drug delivery tool include simple and organic solvent-free production, the flexibility of incorporating both hydrophobic and hydrophilic molecules with high loading, enhanced stability during storage and in physiological environments, the practicability of large-scale preparation and sterilization, controlled drug release, and negligible cytotoxicity [39]. Numerous SLN-based formulations have shown increased efficacy against various tumors [40]. 

Linalool-containing SLN formulations were prepared using cetyl esters, cetyl palmitate, or myristyl myristate using Pluronic^®^F68 as a surfactant [41]. The spherical SLNs displayed mean diameters of 90–130 nm, narrow size distribution, near neutral zeta potential (i.e., −4.0 mV), and high encapsulation efficiency (more than 80%). The developed formulations, especially SLN of myristyl myristate, demonstrated superior in vitro cytotoxicity against HepG2 human hepatocellular carcinoma and A549 human lung adenocarcinoma cells in a dose-dependent manner. Similarly, SLN-encapsulated curcumin exhibited higher cytotoxicity against SKBR3 human breast cancer cells than the free drug [42].

Like liposomes, SLNs have also been conjugated with tumor-specific ligands for active targeting. An arginine–glycine–aspartic acid (RGD) peptide-modified pH-sensitive SLN formulation of doxorubicin was formulated to overcome multidrug resistance in breast cancer [43]. The resulting nanocarriers have a size of 96.3 nm with narrow size distribution and zeta potential of 35.6 mV. The drug loading was found to be 9.8%, with a very high encapsulation efficiency (98.5%). The in vitro drug release at pH 5 medium showed a biphasic release profile with an initial burst of 39% of the drug in the first 8 h, followed by a sustained release until 96 h. However, at physiological (pH 7.4) or higher pH (pH 9), the drug release rate was relatively low compared to pH 5. Furthermore, the RGD-modified SLN formulation of doxorubicin exhibited 5.58-fold higher AUC than the doxorubicin solution. Most importantly, doxorubicin-loaded targeted SLNs showed higher tumor inhibition than free doxorubicin and nontargeted doxorubicin SLNs.

#### 2.1.3. Nanostructured Lipid Carriers (NLCs)

NLCs are second-generation lipid nanoparticles developed to alleviate the drawbacks of SLNs, such as poor drug loading capacity, polymorphic transitions, lipid crystallization with time, and drug leakage during storage [39,44]. Generally, NLCs consist of solid and liquid lipids, surfactants, and other components, including co-surfactants and counter-ions [45]. The solid lipid matrix is immersed in a liquid lipid phase. The incorporation of liquid lipids causes the alteration of the solid lipid matrix from a highly ordered crystalline phase to an imperfect crystalline lattice, which improves drug loading and prevents drug leakage [46,47].

Like SLNs, the most frequently used solid lipids in NLCs are triglycerides, partial glycerides, fatty acids, steroids, and waxes [48]. The commonly used liquid lipids contain digestible natural oils (e.g., corn oil, soybean oil, safflower oil, olive oil, coconut oil, and palm oil), medium-chain triglycerides (e.g., glyceryl tricaprate and glyceryl tricaprylate), fatty acid (e.g., oleic acid, linoleic acid, and capric acid), Cetiol V, Miglyol 812, paraffin oil, isopropyl myristate, squalene, and vitamin [45,48]. A large variety of surfactants and their mixtures have been used to enhance NLC stability in the aqueous phase. However, the frequently used surfactants are Tween (e.g., Tween 20, Tween 40, and Tween 80), poloxamer (e.g., Pluronic F68 and Pluronic F127), Solutol HS15, polyvinyl alcohol, sodium salts of oleic, deoxycholic, and glycolic acids, polyglycerol methyl glucose distearate, TegoCare 450, egg lecithin, and soya lecithin [45]. It has been reported that the stability, crystallinity, and toxicity of NLCs are strongly influenced by surfactant types [49]. 

A study compared the antitumor efficacy of doxorubicin-encapsulated NLCs versus liposomes against a 4T1 breast cancer animal model [50]. Liposome-encapsulated and free doxorubicin showed no differences in mean tumor volume; however, NLC-encapsulated doxorubicin was the most effective in reducing tumor development. Furthermore, both NLCs and liposomes demonstrated the ability to delay the onset of lung metastases.

**Figure 3:**
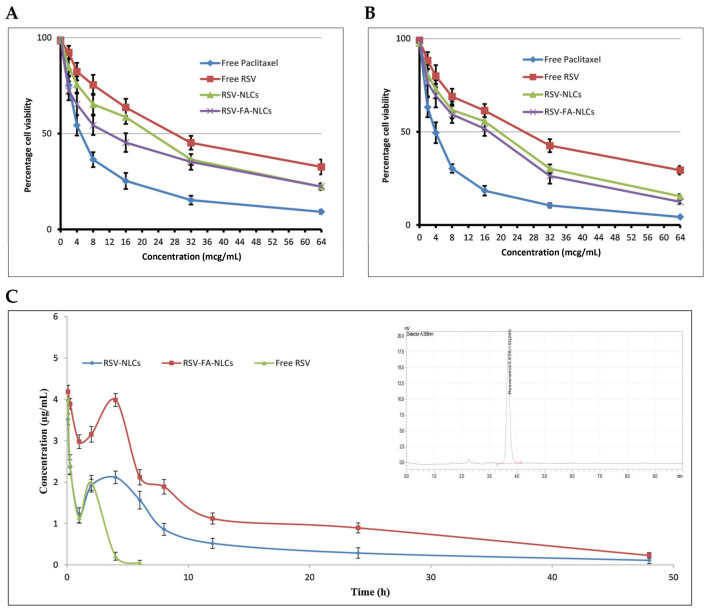
The in vitro cell viability of (**A**) MCF-7 and (**B**) A549 cells at various drug concentrations of the drug after 72 h treatment. (**C**) Plasma concentration–time profile of resveratrol (RSV) in rats with intravenously administered free RSV, RSV-NLCs and RSV-FA-NLCs; inset shows representative HPLC chromatogram of RSV in rat plasma. Source: reprinted from Poonia, N.; Kaur Narang, J.; Lather, V.; Beg, S.; Sharma, T.; Singh, B.; Pandita, D., Resveratrol loaded functionalized nanostructured lipid carriers for breast cancer targeting: Systematic development, characterization and pharmacokinetic evaluation. Colloids and Surfaces B: Biointerfaces 2019, 181, 756–766 [53]. With permission from Elsevier.

Recently, NLCs have gained increasing attention as a potential drug delivery tool for cancer therapy [16,51]. Resveratrol, a naturally occurring nonflavonoid polyphenol, inhibits proliferation, metastasis, and invasion in multiple cancer cell lines by interacting with several molecular targets, such as P53, MAPK, caspases-3, 7, 8, 9, VEGF, and MMP-2 [52]. Nevertheless, the in vivo application of resveratrol is restricted due to its low aqueous solubility, photostability, and substantial first-pass metabolism. An NLC-based parenteral formulation of resveratrol was developed for its efficient delivery to breast cancer cells [53]. The optimized resveratrol-loaded NLCs (RSV-NLCs) showed a size of 88.3 ± 3.1 nm with an entrapment efficiency of 88.0 ± 2.6%. The optimized NLCs were conjugated with folic acid to target folate receptors overexpressed in breast cancer cells. Folic-acid-modified NLCs (RSV-FA-NLCs) showed significantly higher toxicity against MCF-7 folate-receptor-positive human breast cancer cells than unmodified NLC and free resveratrol (Figure 3). In A549 cells, with barely any expression of folate receptors, its cytotoxic effects were less pronounced. Interestingly, folate-conjugated NLCs showed a superior pharmacokinetic profile (t_1/2_: 12.04 h and AUC: 57.92 μg/mL·h) when compared to unmodified NLCs (t_1/2_: 10.38 h and AUC: 27.11 ± 3.92 μg/mL·h) and free resveratrol (t_1/2_: 0.98 h, AUC: 6.37 ± 1.16 μg/mL·h).

A brief list of actively targeted lipid-based formulations with their targeting ligands is enumerated in Table 1.

### 2.2. Inorganic Nanocarriers

Inorganic nanocarriers are made of metal (e.g., gold and silver), metal oxide (e.g., iron oxide, titanium oxide, copper oxide, and zinc oxide), mesoporous silica, graphene oxide, carbon nanotube, and black phosphorous [64,65,66]. These nanocarriers can exist as nanoshells, nanorods, nanocages, nanostars, and nanospheres. The main advantage of inorganic nanoparticles is their high stability compared to organic materials [67]. They can overcome instability issues, such as easy oxidation and hydrolysis, encountered by lipid-based nanocarriers. Additionally, drug molecules can easily leak from lipid-based nanocarriers, making inorganic nanocarriers a potential alternative for drug delivery [65]. The other significant advantages of inorganic nanocarriers include diverse surface chemistry, controllable structures, and unique magnetic, electrical, and optical properties [68,69]. Therefore, besides their drug delivery potential, many inorganic nanoparticles are also used in PDT, PTT, and hyperthermia therapy [70]. Although they offer many potential advantages, they usually suffer from low biocompatibility and biodegradability.

Among various inorganic nanocarriers, iron nanoparticles (FeNPs), gold nanoparticles (AuNPs), carbon nanotubes (CNTs), mesoporous silica nanoparticles (MSNs), and graphene oxide nanoparticles have been extensively investigated for cancer diagnostic and therapeutic purposes.

#### 2.2.1. Iron Nanoparticles (FeNPs)

Over the years, iron nanoparticles (FeNPs) have been widely investigated for diagnostic, therapeutic, and theranostic purposes. FeNPs can be synthesized via physical, chemical, and biological techniques, but the chemical method, particularly coprecipitation, is the most frequently used procedure [71,72,73]. The superparamagnetic properties of iron oxide allow its application as a contrast agent for MRI and magnetic particle imaging (MPI) [74]. While the therapeutic applications of FeNPs include iron supplementation, magnetic hyperthermia, macrophage polarization, and magnetic drug targeting [72]. Nevertheless, the magnetic properties of FeNPs are strongly influenced by their composition and morphology. Therefore, selecting the proper synthesis method is crucial to formulate FeNPs with suitable size, shape, and crystallinity. 

A few potential disadvantages of magnetic nanoparticles associated with their drug delivery application include the difficulty in sustaining efficacy in the targeted tissue after removing the external magnetic field. Moreover, the efficient tumoral distribution of nanoparticles under in vivo conditions is challenging due to its three-dimensional structure [75].

Doxorubicin is a topoisomerase 2 enzyme inhibitor showing promising cytotoxicity against many cancers. However, the effectiveness of doxorubicin in glioblastoma multiforme (GBM) is limited mainly due to its inadequate distribution across the blood–brain barrier. Thus, doxorubicin-loaded trimethoxysilylpropyl-ethylenediamine triacetic acid (EDT)-stabilized biocompatible FeNPs were prepared to enhance their efficacy against GBM [76]. Doxorubicin release under in vitro conditions was finished in 4 days, with a quicker drug release at the acidic pH. The nanoparticle-encapsulated doxorubicin has shown 2.8-fold higher cellular uptake in human U251 human malignant glioma cells than free doxorubicin, leading to superior in vitro cytotoxicity. Furthermore, treatment with nanoformulation significantly downregulated TOP II, Ku70, and MiR-155 oncogene, while concurrently upregulating caspase 3 and tumor suppressor genes p53, MEG3, and GAS5. Most importantly, the application of external magnetic fields further enhanced the permeability and cytotoxicity of nanoparticles in the multidrug-resistant protein 1 transfected Madin–Darby canine kidney cells (MDCK-MDR1)–U251 coculture model.

The higher surface energy, stronger electrostatic interactions, and sensitivity to oxidation make naked superparamagnetic iron oxide nanoparticles (SPIONs) prone to self-agglomeration, limiting their in vivo performance. Surfactant-based surface modification of SPION is one of the potential strategies for improving colloidal stability. However, excessive surfactant addition to nanoparticle formulations may cause particle agglomeration by charge neutralization and bridging. In order to avoid nanoparticle bridging and instability, the quantity of surfactant must be precisely adjusted, resulting in regulated particle size and enhanced dispersion. Furthermore, rapid opsonization and subsequent clearance by the RES is another obstacle to in vivo application of SPION. Numerous materials, such as PEG [77], polylactic acid (PLA) [78], poly lactic-co-glycolic acid (PLGA) [79], chitosan [80], casein [81], and polycaprolactone [82], have been used to coat FeNPs that confer stealth properties and make nanoparticles unrecognizable to the immune system.

Several studies have demonstrated that tyrosine kinase-like orphan receptor (ROR1) is known to overexpress in triple-negative breast cancer (TNBC) cells and is associated with increased tumor cell proliferation [83,84]. Therefore, small interfering RNA (siRNA)-based gene therapy against ROR1 could be a potential treatment strategy for TNBC. However, systemic siRNA delivery is a significant challenge, since it can be readily degraded under physiological conditions. In the past, gene delivery was primarily achieved through viral vectors, which is associated with multiple detrimental effects, such as immunogenicity, inflammation, and insertional mutagenesis [85]. Thus, various lipid-, polymer-, and metal-based nonviral delivery systems are presently developed for targeted gene therapy. The superparamagnetic properties of FeNPs can allow their organ-specific delivery by applying a magnetic field externally. Silk sericin-coated FeNPs (SS-FeNPs) were recently synthesized for tumor-targeted delivery of ROR1 siRNA to treat TNBC [86]. Sericin coating reduces the innate toxicity and immune response caused by FeNPs. SS-FeNPs were further coated with polyethyleneimine (PEI) to impart a positive surface charge to the nanoparticles for siRNA conjugation. The apoptosis study revealed a significantly higher number of apoptotic cells in the ROR1-siRNA-FeNPs treated group than in the control and free ROR1 siRNA. Superior FeNP accumulation in the breast tumor region of BALB/c mice was observed when guided via the magnetic field. In addition, reduced tumor growth was observed in ROR1-siRNA-FENPs-treated mice, along with enhanced necrosis as compared to controls.

**Figure 4:**
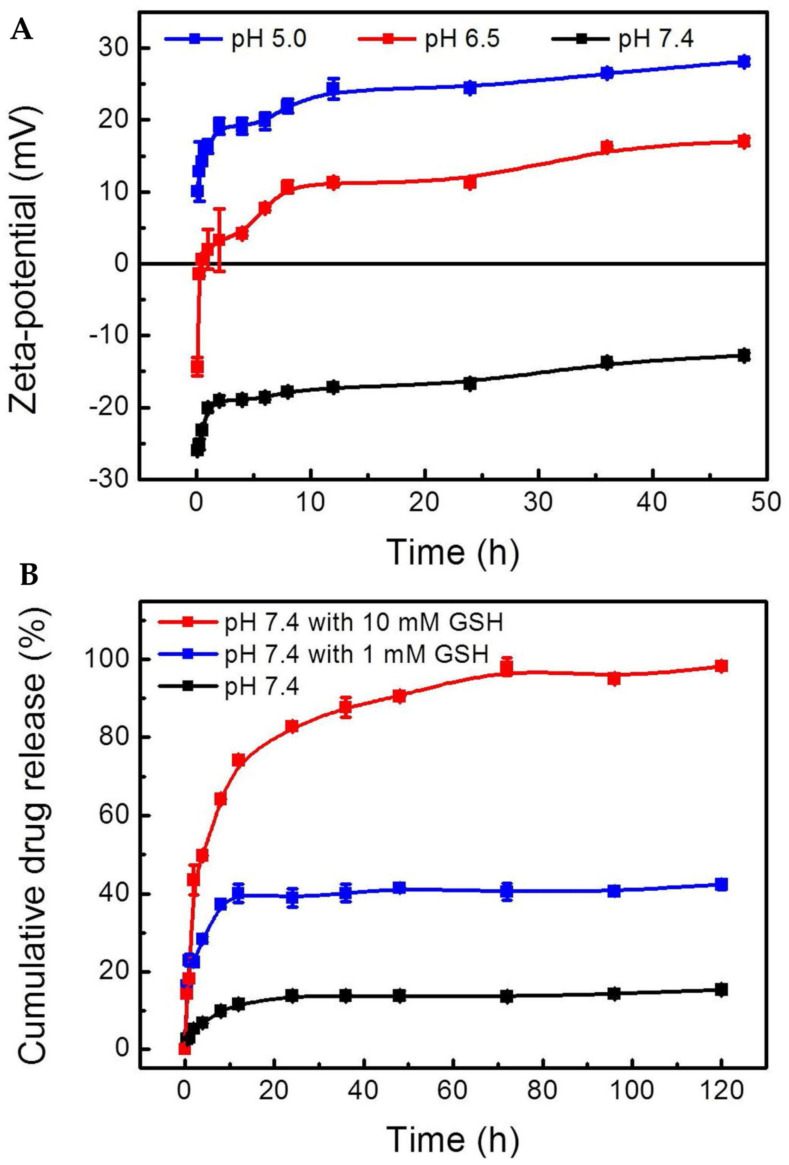
(**A**) Zeta potential as a function of different pH (pH 5.0, 6.5, and 7.4) for DOX@MMSN−SS−PEI−cit. (**B**) Cumulative release of DOX from DOX@MMSN−SS−PEI−cit nanoplatform in PBS (pH 7.4) with different GSH concentrations (0, 1, and 10 mM) in shaking table at 37 °C. Data are shown as mean ± SD, *n* = 3 per treatment. Source: reprinted from Wan, L.; Chen, Z.; Deng, Y.; Liao, T.; Kuang, Y.; Liu, J.; Duan, J.; Xu, Z.; Jiang, B.; Li, C., A novel intratumoral pH/redox-dual-responsive nanoplatform for cancer MR imaging and therapy. Journal of Colloid and Interface Science 2020, 573, 263–277 [87]. With permission from Elsevier.

FeNPs can also be integrated with other nanoplatforms to enable their theranostic applications. With this aim, a monodisperse mesoporous silica-coated multifunctional FeNPs nanoplatform (DOX@MMSN-SS-PEI-cit) was designed to diagnose and treat cancer [87]. Monodisperse mesoporous silica nanoparticles (MMSN) were initially conjugated with branched PEI via disulfide linkages, and citraconic anhydride was further coupled to PEI. Doxorubicin was trapped in mesoporous nanoparticles due to their interaction with PEI. At physiological conditions (pH 7.4), these nanocarriers possess a negative surface charge, which helps to avoid undesired serum protein adsorption and subsequent clearance by the RES. In slightly acidic TME, a negative-to-positive charge reversal occurs due to the hydrolysis of amide bonds, which promotes endosomal escape of the resulting nanocarrier (Figure 4A). The disulfide bonds between MMSNs and PEI can break in a highly reductive environment in the cancer cells, leading to rapid drug release. In vitro release study revealed 98.1 ± 2.22% of doxorubicin released within 72 h in 10 mM glutathione (GSH)-containing media, whereas only 42.4 ± 1.21% drug was released in the presence of 1 mM GSH, and even went further down to 15.3 ± 0.54% in GSH-free media (Figure 4B). Higher uptake of DOX@MMSN-SS-PEI-cit by 4T1 cells was observed at pH 6.5 compared to pH 7.4. The DOX@MMSN-SS-PEI-cit nanocarriers not only helped in MRI imaging of the tumor tissue, but improved tumor-targeted delivery of doxorubicin, resulting in better tumor growth inhibition than the free doxorubicin-treated animals.

In addition to their drug delivery potential, drug-free FeNPs can be extensively used in cancer therapy due to their cell-killing properties via the ferroptosis pathway, a nonapoptotic mechanism. Ferroptosis-inducing factors, such as iron accumulation in a cell, directly or indirectly influence glutathione peroxidase, which decreases antioxidant capacity, aggravates lipid ROS in cells, and ultimately causes oxidative cell death [88]. FeNPs coated with gallic and polyacrylic acids have shown cytotoxic effects against U87MG and U373MG human glioblastoma cell lines, HT1080 human fibrosarcoma cell line, IMR32 human neuroblastoma cell line, and HT22 mouse hippocampal neuronal cell line [89]. Several studies have shown that ferroptosis increases chemotherapeutics’ sensitivity, especially to chemotherapeutic resistance cancer cells [90].

#### 2.2.2. Gold Nanoparticles (AuNPs)

Due to their distinct physicochemical, optical, and electronic properties, AuNPs have been extensively investigated for cancer diagnosis and therapy [91]. Other advantages of AuNPs as drug carriers include their excellent biosafety profile, controlled dispersion, enhanced stability, high surface area for loading drugs, and ease of surface functionalization [92]. Furthermore, they can be formulated into several shapes, such as nanorods, nanocages, hollow nanospheres, nanowires, nanoboxes, and nanostars, each having unique properties, behavior, and applications. AuNPs can be made of either pure gold, combined with additional materials, or doped with other metals to create novel hybrid materials that can be further capped, functionalized, or conjugated with pharmaceuticals or biological molecules for cell targeting and drug administration [93].

Numerous chemical, thermal, physical, electrochemical, biological, or hybrid methods have been used for AuNP synthesis [94,95]. The most widely used chemical procedure is the Turkevich method, which involves the reduction of [AuCl4]^−^ in an aqueous medium using a reducing agent (e.g., tannic acid, ascorbic acid, or citrate) [96]. On the other hand, the most commonly used physical method includes applying radiation and laser ablation [95]. Generally, microwave, ultraviolet, or gamma irradiation is used for AuNP synthesis, providing heat and reducing conditions. In contrast, laser ablation emits particular wavelengths that induce the production of AuNPs. Both physical and chemical AuNPs production processes rely on the elevated temperature, pressure, and exposure to toxic chemicals that limits their applications due to the chances of increasing adverse effects [97]. Thus, researchers nowadays opt for biological methods involving microalgae, bacteria, fungi, and plants to reduce metal salts into stable and biocompatible metals [98].

The unique optical properties of AuNPs enable them to efficiently absorb and scatter light. Exposure of metal nanoparticle to electromagnetic radiation causes conduction electrons on their surface to oscillate collectively due to resonant interaction with the input electromagnetic field, which is called surface plasmon resonance (SPR). The SPR effect of AuNPs is invariably greater than those of non-plasmonic nanoparticles of the same size [99]. The SPR effect of AuNPs is strongly dictated by their size, shape, composition, and concentration [99]. In some cases, particularly shaped AuNPs can capture photons multiple folds higher than photothermal dyes, making them a suitable candidate for PTT. Further, the resonant frequency of the AuNPs can be modulated by altering their size and shape, which allows researchers to use wavelengths within the “biological window (650–1100 nm)” with the lowest impact on blood and other tissues [100].

NIR has been frequently used in PTT-mediated tissue ablation due to its enhanced tissue penetration. It has been reported that the PTT efficacy of AuNPs, especially for deep tissue cancers, is strongly governed by their NIR absorption capacity [101]. However, the size of AuNPs must be greater than 100 nm to obtain enhanced absorptivity for NIR, which exhibits toxic effects due to low excretion and possible accumulation in the body [102]. To evade such conditions, TME-responsive AuNPs have been formulated where small-size AuNPs aggregate at low pH, providing larger particle size for better NIR absorption, and dissemble at physiological pH [103]. The pH responsiveness was achieved by adding a layer of single-stranded DNA (ssDNA) and cytochrome C on the surface of AuNPs. The optimum ratio was 1:400:1000 for AuNPs, ssDNA, and cytochrome, respectively. It was observed that CytC/ssDNA-AuNPs formed a cluster via aggregation at pH 5.5, which was similar to cancer cells’ pH. A decrease in pH leads to a decrease in zeta potential of CytC/ssDNA-AuNPs, increasing the nanoparticle size via electrostatic clustering. This aggregation could be reversed by exposing nanoparticles to pH 7.4, similar to physiological conditions. The low-pH-induced aggregation of AuNPs leads to a red shift of plasmonic absorption peak, conferring greater photothermal effects at acidic pH than at physiological pH. It was observed that the temperature of cell culture media containing CytC/ssDNA-AuNPs at pH 5.5 elevated by 30 °C or more upon NIR irradiation, while only a 9 to 12 °C increase was evident for pH 7.4 culture media. In vitro PTT study exhibited the superior cytotoxicity of CytC/ssDNA-AuNPs against B16F10 melanoma than non-pH-responsive particles without affecting healthy cells. Nanoparticle-mediated PTT was also achieved using NIR-activated fluorouracil–gold nanoparticle complexes, which showed significant antitumor efficacy in colon cancer peritoneal metastasis [104]. 

AuNPs have also been widely used as drug delivery platforms since their surface properties can be easily modified to bind with various therapeutic agents to improve tumor targeting. For instance, eugenol-conjugated AuNPs were studied for potential cancer therapy [105]. The eugenol-conjugated AuNPs demonstrated higher toxicity than free eugenol against PC-3 human prostate cancer and PANC-1 human pancreatic ductal adenocarcinoma cells, indicating that encapsulation of clove phytochemical into AuNPs leads to improved pharmacological potential and bioavailability. In another study, hyaluronic-acid-conjugated dendrimer-encapsulated AuNPs were used for tumor-targeted delivery of doxorubicin [106]. Nanoparticle-encapsulated doxorubicin showed fourfold higher growth inhibition of SK-OV-3 human ovarian cancer cell xenograft tumor than free doxorubicin. 

Additionally, AuNPs can be used to deliver chemotherapeutics and si RNA simultaneously. AuNPs loaded with doxorubicin and Bcl-2 siRNA (Dox-Bcl2-AuNPs) were evaluated for antitumor efficacy on TNBC cells [107]. The 3′-end of Bcl-2 siRNA was attached to the AuNPs surface via thiol conjugation, whereas doxorubicin was directly connected to siRNA via intercalation. The coloaded nanocarriers resulted in a 40% decrease in Bcl-2 expression when incubated with 50 nM siRNA. The Dox-Bcl2-AuNPs not only significantly reduced cell proliferation compared to free doxorubicin, but also inhibited cell migration.

#### 2.2.3. Mesoporous Silica Nanoparticles (MSNs)

MSNs serve as multifunctional nanocarriers for cancer management due to their capability of controlled drug release, tunable pore size, biocompatibility, and ability to transform crystalline drugs to their amorphous state [108,109]. MSNs can encapsulate a large amount of chemotherapeutic agents, biological macromolecules, and multiple metal species due to their rigid framework and large internal pore. For instance, in vivo applications of paclitaxel and camptothecin are often limited due to their poor aqueous solubility. Using MSNs as drug carriers significantly improved the solubility of paclitaxel and camptothecin, which ultimately increased their cytotoxicity by 4.3-fold for paclitaxel against HepG2 cells and ~86% for camptothecin against Capan-1 human pancreatic adenocarcinoma cells [110,111]. 

**Figure 5:**
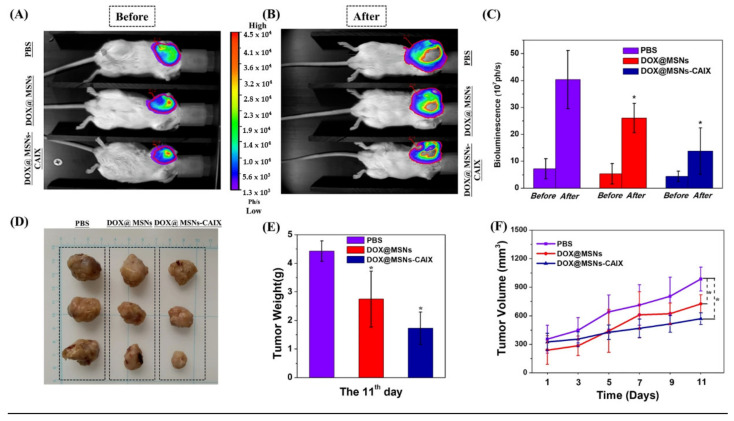
4T1-Luc tumor-bearing mice treated with different samples (PBS, DOX@MSNs, and DOX@MSNs-CAIX) for 11 days. The tumors’ bioluminescence imaging before (**A**) and after (**B**) intervention with the above samples. (**C**) The tumors’ bioluminescence intensity before (the 0th day) and after (the 11th day) intervention with the above samples. (**D**) The tumors’ photographs from the scarified mice. (**E**) The final average tumor weight. (**F**) The variation curves of average tumor volume. (* *p* < 0.05 as compared with PBS group.) Source: reprinted from Chen, M.; Hu, J.; Wang, L.; Li, Y.; Zhu, C.; Chen, C.; Shi, M.; Ju, Z.; Cao, X.; Zhang, Z., Targeted and redox-responsive drug delivery systems based on carbonic anhydrase IX-decorated mesoporous silica nanoparticles for cancer therapy. Scientific Reports 2020, 10, (1), 14447 [112] under an open access Creative Commons CC BY 4.0 license.

Additionally, numerous ligand molecules can be conjugated to the MSN surface for active targeting. An antibody-targeted and redox-responsive doxorubicin-loaded MSN-based drug delivery system (DOX@MSNs-CAIX) was designed by conjugating anti-carbonic anhydrase IX antibody (CAIX) on the MSNs surface via disulfide linkages [112]. CAIX is highly expressed in solid tumor tissue, such as lungs, esophagus, head, breast, bladder, uterine cervix, and kidney carcinoma, compared to healthy tissue. Therefore, MSNs functionalized with anti-CAIX antibodies to improve their tumor-targeting potential to 4T1 cells. Under in vitro conditions, DOX@MSNs-CAIX showed redox-responsive drug release in the presence of GSH due to the cleavage of disulfide linkers in MSNs. Furthermore, higher drug release was also observed with a decrease in the pH of the release medium. DOX@MSNs-CAIX showed efficient tumor accumulation and enhanced antitumor efficacy in 4T1 tumor-bearing mice (Figure 5).

Similarly, pH-responsive folic-acid-decorated MSNPs (MSN-COOH-Tet-HBP-FA) were used for tumor-specific delivery of a chemotherapeutic agent, tetrandrine [113]. The use of amino-terminated hyperbranched polymer imparts pH-responsive properties to this system. MSN-COOH-Tet-HBP-FA demonstrated pH-dependent drug release profiles with negligible release within 20 h in a typical physiological environment. Moreover, the synthesized nanoparticles showed greater uptake and higher cytotoxicity against HeLa and A549 cells.

#### 2.2.4. Carbon Nanotubes (CNTs)

Since their discovery in 1991 by Sumio Iijima, CNTs have opened a new door for research [114]. CNTs comprise single or multiple concentric graphene sheets wrapped into cylinders with a diameter of 0.4–100 nm, although their length can be up to a few micrometers. CNTs can be classified into single-walled carbon nanotubes (SWCNTs) and multi-walled carbon nanotubes (MWCNTs), depending on the number of graphene sheets. The excellent structural features of CNTs make them suitable for use in various industries, including the biological, pharmaceutical, electrical, and material sectors [115,116,117]. SWCNTs are smaller in size, flexible, and provide imaging features. In contrast, MWCNTs consist of multiple graphene sheets forming a complex network of graphene cylinders and offer a high surface area suitable for efficient drug loading [118].

Due to their lipophilic nature, unmodified CNTs are very problematic to disperse in aqueous media and are highly toxic, restricting their in vivo applications. However, CNTs can be readily functionalized by several covalent and noncovalent modifications to enhance their aqueous solubility and bioavailability [119,120,121]. Surface PEGylation of CNTs is a promising strategy to improve their biocompatibility and biological half-life. Zhao et al. [115] formulated PEGylated MWCNTs for tumor-targeted intracellular triggered release of doxorubicin. The longer pristine MWCNTs showed apparent cytotoxicity, while the PEGylated MWCNTs (size ≤300 nm) displayed improved cytocompatibility. The optimized PEGylated MWCNTs exhibited high drug-loading capacity and enhanced toxicity against HepG2 cells. 

**Figure 6:**
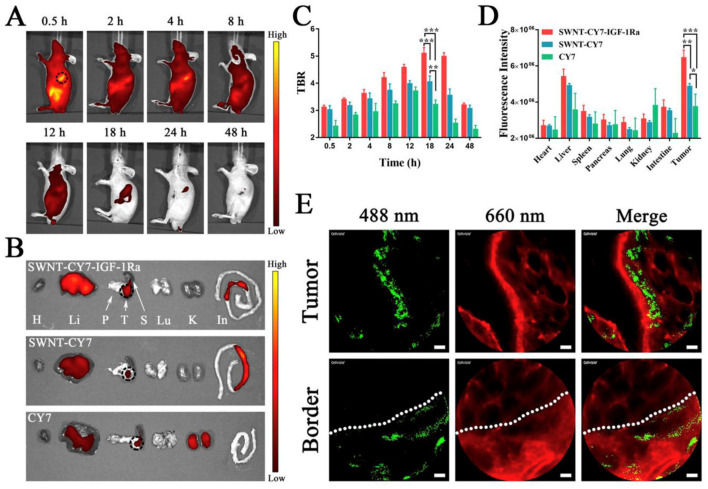
Distribution of nanoprobes in vivo. (**A**) In vivo continuous observations (48 h) of mice administered SWNT-CY7-IGF-1Ra via the tail vein. The black dotted circle represents the location of the pancreatic carcinoma in situ. (**B**) Ex vivo imaging of tumor and major organs. H: heart. Li: liver. P: pancreas. T: tumor. S: spleen. Lu: lung. K: kidney. In: intestine. (**C**) Comparison of TBR (TBR = average fluorescence intensity of the tumor area/average fluorescence intensity, with the ear as the background area) profiles of the nanoprobes. The peak was at 18 h post-injection, which implied an optimal experimental window. (**D**) Fluorescence intensity of different tissues. Data represent mean ± SD of triplicate experiments. (**E**) The accumulation of as-prepared nanotubes along the tumor blood vessels and at the normal–tumor tissue junction at 18 h post-injection. The as-prepared nanotubes appear as green fluorescent dots in 488 nm and the blood vessels are shown as red fluorescent regions in 660 nm. The scale bar is 20 μm. * *p* < 0.05, ** *p* < 0.01, *** *p* < 0.001. Source: Lu, G.-H.; Shang, W.-T.; Deng, H.; Han, Z.-Y.; Hu, M.; Liang, X.-Y.; Fang, C.-H.; Zhu, X.-H.; Fan, Y.-F.; Tian, J., Targeting carbon nanotubes based on IGF-1R for photothermal therapy of orthotopic pancreatic cancer guided by optical imaging. Biomaterials 2019, 195, 13–22 [124]. With permission from Elsevier.

Acid-functionalized MWCNTs were developed for breast cancer treatment [122]. The combined treatment with acid-functionalized MWCNTs and local hyperthermia led to complete tumor eradication in EMT6 tumor-bearing mice, with no animal death during the 50-day study period. There was an increased expression of Hsp70 in hyperthermia-treated mice. Furthermore, an increased immune response was observed in the tumor site for the combined treatment group. MWCNTs were also conjugated with a Pgp-specific antibody to selectively target drug-resistant cells, followed by local tumor ablation with photoirradiation [123]. In another study, Cy7 and IGF-1R antibody-conjugated SWCNTs (SWNT-CY7-IGF1-Ra) were synthesized to allow imaging-guided targeted delivery to IGF-1R receptor overexpressing pancreatic cancer cells for cytotoxic PTT [124]. Biodistribution of SWNT-CY7-IGF1-Ra was performed in BXPC-3 tumor-bearing mice following tail vein injection and compared with SWNT-CY7 and CY7 nanoprobes. The CY7-associated near-infrared (NIR) fluorescence intensity was recorded over multiple time points using IVIS spectrum small animal imager. The SWNT-CY7-IGF1-Ra nanoprobe was initially distributed into the entire body but retained only in the tumor site for a prolonged period, and associated fluorescence was visible in the tumor even after 48 h post-injection (Figure 6A). The biodistribution of various nanoprobes was further quantitatively evaluated in the isolated tissues 24 h post-injection. The kidneys mainly metabolized the free CY7 dye, while SWNT-CY7-IGF-1Ra and SWNT-CY7 were primarily metabolized by the liver and intestine (Figure 6B,D). Nevertheless, SWNT-CY7-IGF1-Ra had shown consistently higher tumor accumulation over the entire period than other nanoprobes (Figure 6C). Interestingly, the nanotube tracking has shown substantial SWNT-CY7-IGF1-Ra accumulation (green spots) on the tumor but not in the normal tissue (Figure 6E).

#### 2.2.5. Graphene Oxide Nanoparticles (GONPs)

Graphene is a key constituent of different graphitic materials, which may be packed into spherical structures (0D fullerenes), wrapped into 1D structures (CNTs), or stacked into 3D-layered systems (graphite) [125,126]. It has a planner structure where sp^2^ hybridized carbon atoms are connected to form a hexagonal lattice. Graphene and its derivatives have several attractive properties, including high drug loading capacity, good biocompatibility, adjustable amphiphilicity, and tunable size and shape, making them suitable for various cancer-related applications (e.g., including biosensing, drug transport, and photothermal treatment) [126,127]. Graphene also has a high surface area, superb thermal and electrical conductivity, the largest strength-to-mass ratio, and excellent tensile strength [128,129].

Graphene oxide (GO), an oxidized form of graphene, offers distinct advantages for biomedical applications due to its ease of production, higher stability, better aqueous solubility, and superior optical properties. Consequently, numerous GO-based nanomaterials were designed to incorporate GO or its derivatives with chemotherapeutic drugs, antibodies, and nanomaterials [128,130,131]. For instance, multifunctional biocompatible GO-Fe_3_O_4_ conjugates were developed for dual MRI/fluorescence imaging and magnetic targeting [132]. The GO-Fe_3_O_4_ conjugates could load a substantial amount of doxorubicin by noncovalent complexation with a mean diameter of 260 nm. Cancer cells efficiently took up the conjugates and exhibited 2.5 times better efficacy over free doxorubicin. As a result, the conjugates could induce similar cytotoxicities, even at one eighth of the dose of free doxorubicin.

Developing a tumor-targeted delivery platform with improved biodegradability is important for successful chemotherapy. In a study, GONPs were conjugated with N-formyl-methionyl-leucylphenylalanine to target the formyl peptide receptor overexpressing cancer cells, including cervical carcinoma cells [133]. The synthesized hybrid nanocarriers showed excellent biodegradability upon treatment with human myeloperoxidase enzyme. The hybrid nanomaterial itself, but not the pristine GO, was able to induce neutrophil degranulation without any prior activation. Additionally, the hybrid nanomaterial could deliver doxorubicin faster in HeLa human cervical cancer cells and induce a greater extent of apoptosis.

The mitochondrion is a crucial subcellular organelle involved in ATP synthesis and plays a critical role in biosynthesis, metabolism, signaling, protein synthesis, and cell death. Therefore, mitochondria are associated with each step of tumorigenesis, from tumor initiation, progression, survival, and metastasis. Consequently, the mitochondrion has become a novel target for cancer therapy. Nevertheless, targeting mitochondria inside cancer cells has remained a significant challenge. To overcome this issue, PEI-coated self-assembled GONPs (PEI-GTC-NP) containing cisplatin and topotecan were synthesized [134]. After six hours of incubation, nanoparticles were efficiently accumulated in the HeLa cells’ mitochondria, generating transition pores on the mitochondrial outer membrane. PEI-GTC-NP induced mitochondrial damage, producing ROS that ultimately cause cancer cells.

### 2.3. Polymeric Nanoparticles

Polymeric nanocarriers have appeared as a powerful tool for tumor-targeted delivery of chemotherapeutics. As a drug carrier, they offer several advantages, including high entrapment efficiency, improved physiological stability, ease of surface functionalization, ability to scale up, and feasibility of manufacturing under good manufacturing practices [135,136]. The composition and molar ratio of the polymer can also be altered to achieve an optimized degradation rate, regulate drug release, and enhance cellular uptake [137]. Furthermore, polymeric nanoparticles have significant payload flexibility and may be loaded with hydrophobic and hydrophilic molecules, small to large molecules, protein, DNA, and RNA [138,139,140]. Drug molecules can be loaded into nanoparticles via adsorption, encapsulation, and conjugation [141]. Based upon the nanoparticle formulation process, the formed polymeric nanoparticles contain the drug either throughout the polymeric matrix or in the core of the particles. Commonly used polymers for nanoparticle preparation include PLGA, PLA, poly(glycolic acid) (PGA), PCL, PLGA–PEG, and some natural polymers, such as alginate, chitosan, gelatin, and albumin.

PLGA is an FDA-approved polymer widely investigated for biomedical applications [142]. It is composed of a copolymer of lactic acid and glycolic acid synthesized at various ratios [143]. Due to its biocompatibility, biodegradability, low toxicity, and sustained-release profile, multiple studies have used PLGA nanoparticles as drug carriers [144]. PLGA nanoparticles loaded with docetaxel and LY294002 (PI3K/AKT signaling inhibitor) exhibited controlled release of therapeutic agents as well as enhanced accumulation at the tumor site [145]. The nanoparticles showed superior anticancer activity against both the xenograft and orthotopic mouse models of gastric cancer.

Unfortunately, PLGA nanoparticles cannot directly interact with specific receptors or cell surface markers overexpressed on cancer cells, preventing medicines from accumulating in the tumor tissues. Therefore, PLGA nanoparticles were often decorated with ligand molecules targeting the overexpressed receptor on the surface of cancer cells. With this aim, RGD-conjugated PLGA nanoparticles (RGD-PLGA NPs) were developed for controlled and targeted delivery of cisplatin and upconversion nanoparticles to treat lung cancer [146]. These nanoparticles exhibited controlled drug release for up to 72 h. The RGD-conjugated nanoparticles displayed 6.3-fold higher AUC than the marketed Ciszest-50 injection. Moreover, RGD-PLGA NPs demonstrated negligible systemic toxicity, low lung tissue damage, and enhanced biocompatibility.

A further downside of PLGA nanoparticles is very high burst release, which could be potentially hazardous. Several coating agents, including chitosan (CS) and alginate, have been employed to minimize the initial burst release issue. For instance, docetaxel-loaded PLGA nanoparticles were coated with chitosan and folic acid to enhance docetaxel delivery to cancer cells and improve its drug release profile [147]. The in vitro docetaxel release exhibited an inverse relationship with the quantity of folic acid chitosan utilized and the pH of the release medium. Coated nanoparticles enhanced the cytotoxic effect of docetaxel compared to the free drug.

Chitosan is frequently utilized in biomedical science, particularly in cancer therapy, because of its excellent biocompatibility, biodegradability, nontoxicity, and low immunogenicity [144,147]. It contains numerous protonable amino groups throughout its backbone and, because amino groups are protonated, it is more soluble in acidic environments. These amino groups can be modified chemically to improve their solubility, biocompatibility, and targeting ability [148,149]. 

Chitosan nanoparticles were designed for the sustained release of doxorubicin in the breast tumor microenvironment [150]. The nanoparticle surface was PEGylated to improve blood circulation time. The PEGylated nanoparticles were further functionalized with anti-human mammaglobin (Anti-hMAM) and anti-human epidermal growth factor (Anti-HER2) antibodies to improve tumor targeting ability. The antibody-conjugated PEGylated nanoparticles showed superior cytotoxicity towards MCF-7 cells compared to free doxorubicin.

**Figure 7:**
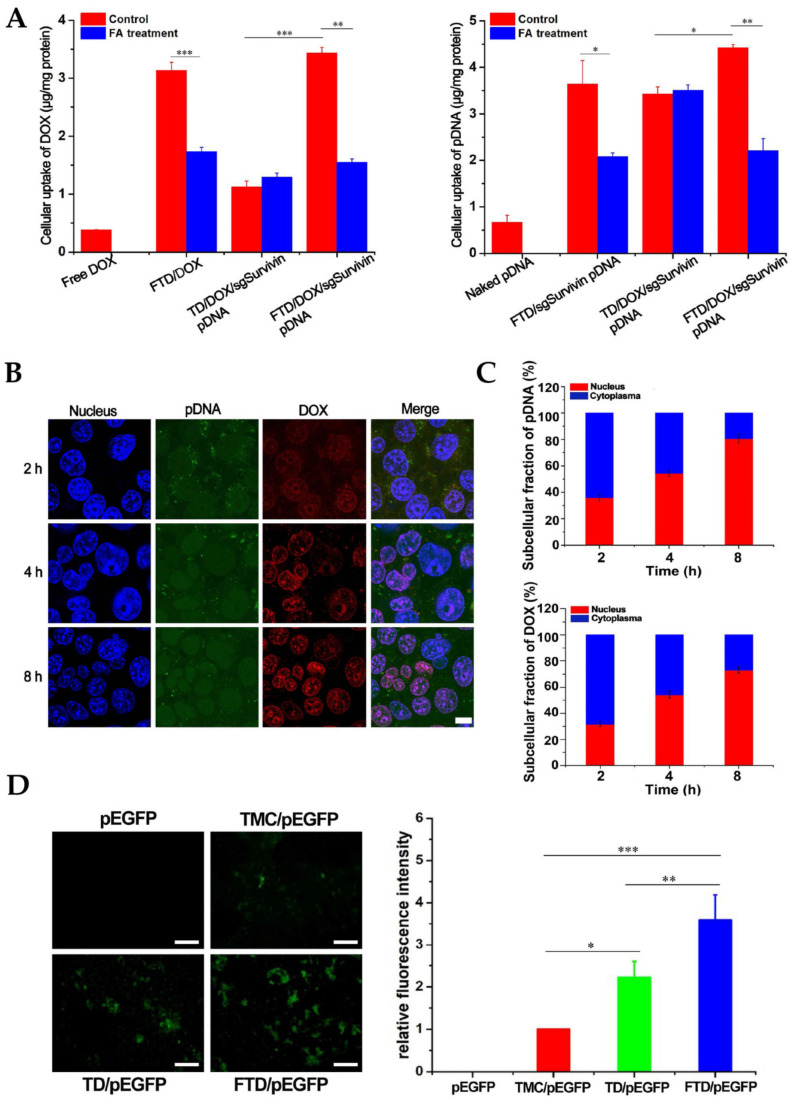
In vitro delivery efficacies of folic acid and 2-(Diisopropylamino) ethyl methacrylate dual-functionalized trimethyl chitosan nanoparticles (FTD NPs). (**A**) Cellular uptake of doxorubicin (DOX) and pDNA in 4T1 cells after 4 h incubation with NPs. Indicated values are mean ± SD (*n* = 3). * *p* < 0.05, ** *p* < 0.01, *** *p* < 0.001. (**B**) CLSM images showing the nuclear transport of FITC-pDNA (green) and DOX (red) loaded into FTD NPs in 4T1 cells after 2, 4, and 8 h incubation. The nuclei were stained with Hoechst 33,258 (blue). Bar represents 10 μm. (**C**) Intracellular distribution of DOX and pDNA in 4T1 cells following treatment with FTD/DOX/sgSurvivin pDNA NPs for 2, 4, and 8 h. Indicated values are mean ± SD (*n* = 3). (**D**) Fluorescence microscope images and relative fluorescence intensity of pEGFP in 4T1 cells transfected for 48 h. Indicated values are mean ± SD (*n* = 3). Bar represents 50 μm. * *p* < 0.05, ** *p* < 0.01, *** *p* < 0.001. Source: reprinted from Li, Q.; Lv, X.; Tang, C.; Yin, C., Co-delivery of doxorubicin and CRISPR/Cas9 or RNAi-expressing plasmid by chitosan-based nanoparticle for cancer therapy. Carbohydrate Polymers 2022, 287, 119315 [151]. With permission from Elsevier.

In another study, folic acid and 2-(Diisopropylamino) ethyl methacrylate (DPA) dual-functionalized trimethyl chitosan nanoparticles (FTD NPs) were formulated for the codelivery of doxorubicin and Survivin shRNA-expressing plasmid (iSur pDNA) or Survivin CRISPR/Cas9-expressing plasmid (sgSurvivin pDNA) [151]. Folic acid modification improved nanoparticle delivery into cancer cells (Figure 7). Because of the pH sensitivity of nanoparticles resulting from DPA conjugation, a fast release of doxorubicin was observed under acidic circumstances. The anticancer efficiency of CRISPR/Cas9 and RNAi-loaded nanoparticles was comparable. However, doxorubicin and sgSurvivin pDNA-loaded nanoparticles were more effective than those of single administration of doxorubicin or sgSurvivin pDNA and equivalent to those of doxorubicin and iSur pDNA-loaded nanoparticles. 

Polymeric micelles are another member of polymeric nanoparticles consisting of a hydrophobic core and a hydrophilic shell produced by the self-assembling of amphiphilic block copolymers in an aqueous solution. The interior hydrophobic core acts as a solubilization depot for poorly water-soluble or hydrophobic agents, increasing their bioavailability. The hydrophilic shell has two functions: longer blood circulation time and increased blood stability. Tumor-specific ligands can be added to polymeric micelles to increase tumor accumulation. As a result, polymeric micelles can play a significant role in delivering hydrophobic medicinal compounds for cancer treatment [152]. 

A polymeric micelle composed of PLGA-PEG-retinoic acid (RA) was developed for the targeted delivery of irinotecan to HT-29 human colorectal and HepG2 cells [153]. Targeted nanomicelles lead to significant cytotoxicity of HepG2 and HT-29 cell lines compared to nontargeted nanomicelles and the free drug. Similarly, micelles of PEG-poly(beta-amino ester) (PBAE)-PEG triblock copolymer were synthesized for pH-dependent delivery of water-insoluble anticancer drug verteporfin [154]. The micelle morphology was also regulated by varying the hydrophobicity of the core PBAE block of the copolymer to avoid macrophage absorption. Micellar formulations showed effective anticancer activity against human TNBC and small cell lung cancer cells. 

PEGylation of nanocarriers increases the circulation time of nanocarriers by preventing them from opsonization. However, PEGylation may often hinder the cellular uptake of nanocarriers. Thus, to enhance the uptake, cRGD peptide ligand-decorated polymeric micelles composed of PEG–poly(l-lysine) (PEG–PLL) block copolymers were synthesized. Antiangiogenic plasmid DNA was incorporated into the central core of polymeric micelles via electrostatic complexation. The resulting nanocarrier showed remarkable transfection efficacy by enhanced uptake via tumor cells. The in vivo treatment exhibited the formulation effectively inhibited BxPC3 tumor growth [155].

Oncoproteins, such as transcription factors, were also targeted using antibody-loaded polymeric micelle for antitumor activity. The pH-responsive polymeric micelles loaded with anti-c-Myc antibodies were capable of escaping from hyperacidified endo/lysosomes of cancer cells while preventing their release from endosomes of healthy cells. Consequently, antibody-loaded micelles efficiently suppressed c-MYC in tumor tissue, thereby inhibiting tumor growth [156]. 

Dendrimers are a distinct class of polymers that have also been frequently used in cancer nanomedicine. They are highly organized, branching polymeric macromolecules with distinct and uniform sizes and shapes [157]. Its fundamental structure consists of three primary parts: a central core, repeated branching units, and terminal groups that offer tunable surface characteristics. Chemotherapeutic agents can be loaded onto dendrimers via encapsulation into the dendrimer core or binding to the surface [158,159]. Numerous peripheral functional moieties improve the capacity to load and deliver therapeutic substances. Furthermore, dendrimers’ molecular weight and chemical composition may be carefully controlled by regulating their production, enabling predictable pharmacokinetics and biocompatibility adjustment.

A dendrimer can be built on virtually any form of chemistry, and the nature of that chemistry greatly influences its solubility, degradability, and biological activity [159]. Polyamidoamines (PAMAM) [160], poly-l-lysine (PLL) [161], polyamides (polypeptides) [162], poly (propylene imine) [163], and carbohydrates [164] are some of the most frequently used forms of dendrimers in biological applications. However, PAMAM dendrimers are the most prevalent dendrimer scaffold, commercially available with a wide range of generations and surface properties. 

*N*-acetyl-d-glucosamine (NAG)-conjugated PAMAM dendrimers were prepared to enhance camptothecin delivery to A549 cells expressing lectin receptors and glucose transporters [165]. NAG-coupled dendrimer was 4.5-fold more toxic to A549 cells than the free drug. Unfortunately, under physiological circumstances, these cationic amine groups are detrimental to cells, limiting the in vivo applications of PAMAM. PEGylation has been the simplest and most extensively utilized method for modulating PAMAM surfaces to enhance biocompatibility and systemic circulation time [166,167].

A self-assembling amphiphilic dendrimer was developed that creates supramolecular micelles with a substantial void area [168]. This design allowed a higher amount of chemotherapeutic agent loading with high encapsulation efficiency. The resultant drug-encapsulated nanomicelles can significantly improve drug potency and counteract drug resistance by boosting cellular uptake and minimizing the efflux of the cytotoxic agent.

### 2.4. Biological Nanocarriers

Since 1990, synthetic nanocarriers such as inorganic, lipid-based, and polymeric nanomaterials have been investigated extensively to enhance the therapeutic efficacy of chemotherapeutic agents. Nevertheless, forced premature treatment termination has been brought in many instances due to the stimulation of host immune responses against these nanocarriers. Biological carriers such as exosomes can function as a nanoplatform in overcoming the drawbacks of conventional delivery systems and enabling successful drug delivery with enhanced drug targeting capabilities. Exosomes are membrane-bound extracellular vesicles having a size ranging from 40 to 150 nm [169]. They are released by both healthy as well as cancer cells and are found in different body fluids, such as milk, plasma, amniotic fluid, saliva, urine, cerebrospinal fluid, sputum, and ascites [170]. Exosomes carry critical information about their parent cells in the form of signaling molecules, making them promising biomarkers for early diagnosis and prognosis of cancer and other inflammatory diseases [171,172,173]. For example, exosomes isolated from breast cancer patient serum have been shown to express CD24, indicating exosomal CD24 could serve as a potential marker for breast cancer [174]. 

**Figure 8:**
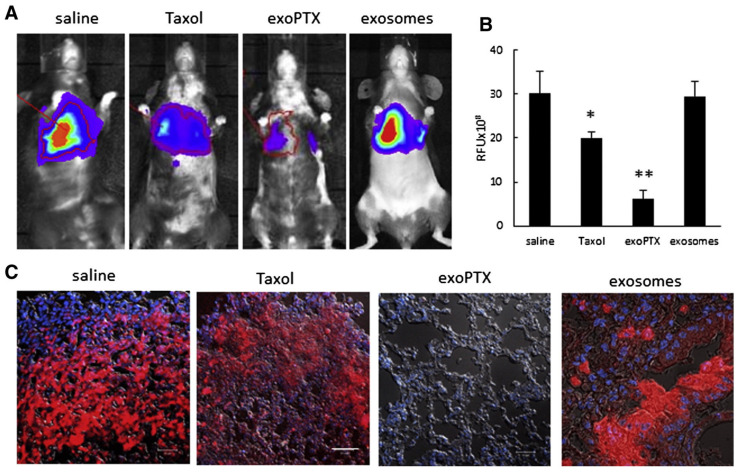
The inhibition of metastases growth in mouse lungs upon exosome-loaded paclitaxel (exoPTX) treatment. C57BL/6 mice were i.v. injected with 8FlmC-FLuc-3LL-M27 (red) cells to establish pulmonary metastases. After 48 h, mice were treated with exoPTX, or Taxol, or saline, or empty sonicated exosomes as a control, and the treatment was repeated every other day, totally, seven times. Representative IVIS images were taken at day 21 (**A**). Statistical significance of metastases levels from IVIS images in lungs of treated animals compared to control mice is shown by asterisk (* *p* < 0.05; ** *p* < 0.005) (**B**). At the endpoint, 21 days later, mice were sacrificed, perfused, and lung slides were examined by confocal microscopy (**C**). The bar: 10 μm. Source: reprinted from Kim, M. S.; Haney, M. J.; Zhao, Y.; Mahajan, V.; Deygen, I.; Klyachko, N. L.; Inskoe, E.; Piroyan, A.; Sokolsky, M.; Okolie, O.; Hingtgen, S. D.; Kabanov, A. V.; Batrakova, E. V., Development of exosome-encapsulated paclitaxel to overcome MDR in cancer cells. Nanomedicine: nanotechnology, biology, and medicine 2016, 12, (3), 655–664 [168]. With permission from Elsevier.

Exosomes convey their instruction to the target cells via surface receptor interaction, membrane fusion, receptor-mediated endocytosis, phagocytosis, and/or micropinocytosis. These strategies effectively allow exosomes to deliver therapeutic agents to targeted tissue and cells. Several techniques, including electroporation, transfection, incubation, extrusion, sonication, thermal shock, freeze–thaw cycles, hypotonic dialysis, and pH gradient method, have been explored to load exosomes with diverse chemotherapeutic drugs and other bioactive compounds [175]. For example, exosome-loaded paclitaxel (exoPTX) was evaluated to overcome multidrug resistance in cancer cells [176]. RAW 264.7 mouse macrophage-derived exosomes were loaded with paclitaxel via incubation at room temperature, electroporation, or mild sonication, and the free drug was removed from the exoPTX by size exclusion chromatography. The anticancer efficacy of exoPTX formulation was studied using multidrug-resistant MDCK-MDR1 cells and their sensitive wild-type counterparts (MDCK-WT) cells. The exoPTX formulation showed greater cytotoxicity against both resistant and sensitive cells than free paclitaxel or Taxol. As expected, MDCK-MDR1 cells were more affected by the exoPTX than the sensitive MDCK-WT cells. The anticancer activity of exoPTX was further evaluated using the murine Lewis Lung Carcinoma pulmonary metastases model. Treatment with exoPTX demonstrated significant (*p* < 0.05) tumor growth inhibition compared to nontreated control and Taxol-treated animals (Figure 8).

In a recent study, an exosome-based bioplatform was developed to reprogram TME and improve anticancer efficacy against pancreatic ductal adenocarcinoma (PDAC) [177]. Bone marrow mesenchymal stem cells (BM-MSC)-derived exosomes were initially loaded with galectin-9 siRNA via electroporation process, while oxaliplatin–maleimide was conjugated to siRNA-loaded exosome surface via maleimide–thiol conjugation reaction. The tumor-homing capability of BM-MSC exosomes resulted in an increased accumulation of therapeutic agents in the PDAC tissue while minimizing systemic toxicity. Further, the exosome-mediated combined treatment exhibited effective antitumor immunity via tumor-suppressive macrophage polarization, cytotoxic T lymphocyte recruitment, and regulatory T cell downregulation and accomplished efficient anticancer efficacy in ANC-02 tumor-bearing mice.

The MSC-derived exosomes have also been used for combined cancer hyperthermia and cytosine deaminase (CD)/5-fluorocytosine (5-FC) prodrug gene therapy [178]. The CD/5-FC system is a well-established gene-directed enzyme/prodrug therapy that converts nontoxic 5-FC into cytotoxic 5-FU [179]. FeNP-loaded exosomes were isolated from MSCs and MSC-expressing yeast CD::uracil phosphoribosyl transferase suicide fusion gene (yCD::UPRT-MSCs) labeled with Venofer (i.e., carbohydrate-coated iron oxide nanoparticles). Both MSCs and yCD::UPRT-MSCs exosomes were efficiently internalized by tumor cells and exhibited hyperthermia-induced apoptosis upon exposure to the alternating magnetic field (AMF). A further improvement in cytotoxicity was observed when AMF-exposed viable tumor cells underwent treatment with yCD::UPRT-MSCs in the presence of 5-FC.

Like other nanocarriers, the tumor-targeting ability of exosomes could also be improved by functionalizing them with tumor-specific ligands. To this end, doxorubicin-loaded milk exosomes (mExo) were functionalized with hyaluronan to facilitate their delivery to CD44-overexpressing cancer cells [180]. Hyaluronan (HA) was initially coupled to amphiphilic DSPE-PEG_2000_ to synthesize DSPE-PEG_2000_-HA conjugate. The incubation of doxorubicin-loaded mExos (mEXO-Dox) with DSPE-PEG_2000_-HA conjugate allows the embedding of hyaluronan onto the phospholipid bilayer of mExo. The hyaluronan functionalized mExo-Dox has shown selective doxorubicin delivery to CD44 overexpressing cancer cells and promoting more significant cell death. 

Along with their drug delivery potential, exosomes can also be used as a theranostic platform. An exosome-based theranostic tool was developed to image and deliver olaparib to hypoxic tumor regions [181]. Exosomes of four different kinds were derived from MDA-MB-231 cells in normoxic or hypoxic conditions and with or without radiation exposure. These exosomes were tagged with 3,3′-Dioctadecyloxacarbocyanine perchlorate (DiO) fluorescent dye to assess their uptake by hypoxic cancer cells. Subsequently, exosomes were loaded with superparamagnetic iron oxide nanoparticles and olaparib. It has been reported that the hypoxia cells preferentially pick up exosomes generated by hypoxic cells in comparison to other exosome formulations. Furthermore, the biodistribution of iron-oxide-labeled exosomes was visualized using MPI. Finally, higher apoptosis and slower tumor progression confirmed the therapeutic effectiveness of olaparib-loaded exosomes.

## 3. Current Status of Cancer Nanomedicine

Current cancer therapy is mainly confined to surgery, radiation, and chemotherapy. All three approaches are limited by the incomplete removal of cancer tissues and the risk of damaging healthy tissues. Nanotechnology provides the tools to precisely and directly target chemotherapies to cancerous cells, guide surgical tumor resection, and improve the therapeutic efficacy of radiation-based and other existing treatment modalities. These may result in a lower risk for the patient and a higher chance of survival.

The first nanotechnology-based cancer drug approved by U.S. FDA is the PEGylated doxorubicin liposomal formulation (Doxil^®^/Caelyx^®^) marketed in 1995. Since that, there has been a significant advancement in developing nanotherapeutic formulations, and several nanomedicines have been approved by FDA and other regulatory agencies for cancer treatment. Liposomes (PEGylated or non-PEGylated), along with other lipid-based nanoparticles, continue to make up a significant percentage of marketed nanotherapeutics. Nevertheless, many other nanoplatforms, such as polymeric, metallic, or protein-based nanoparticles, have also been approved for cancer therapy. 

Despite significant advancement in ligand-based active tumor targeting over the years, most clinical-stage nanoformulations, if not all, still rely on EPR-based tumor accumulation. Table 2 summarizes approved cancer nanotherapeutics with their active pharmaceutical ingredients, manufacturer, and authorized uses. Besides approved nanomedicines, the FDA has granted multiple Investigational New Drug applications for nanoformulations in recent years, allowing clinical studies for lung, breast, gynecological, gastric, pancreatic, lymphoma, central nervous system, and genito-urinary cancer therapies (Table 3). Most of these trials use conventional chemotherapeutics with a previously established nanoplatform.

## 4. Challenges and Future Prospects

We have seen great developments in nanotechnology over the last few decades and our scientific understanding of the mechanisms governing nanoparticle physicochemical properties and their interaction with biological systems has advanced dramatically. Yet, given the speed and scope of nanotechnology research, the use of nanotechnology for cancer diagnosis and treatment is still in the development phase. Several challenges are associated with nanoparticle development, greatly limiting their success in clinical settings.

First, all marketed cancer nanomedicines rely on the EPR effect for passive tumor targeting. Nevertheless, the EPR effect itself is impacted by tumor heterogeneity, resulting in inter-patient and even intra-patient variability. On top of that, some studies suggest that the EPR effect is more prominent in smaller animals than in humans, which may lead to skewed data about the efficacy of a particular therapy. To overcome the issue faced by first-generation nanomedicines (i.e., EPR-based), nanocarriers with advanced tumor-targeting potentials were formulated with the hope of superior clinical outcomes. For instance, several nanoformulations currently undergoing clinical testing seek to improve the effectiveness of cancer treatment through active targeting (e.g., BIND-014: PSMA-directed docetaxel nanoparticle) and stimuli-responsive drug release (e.g., ThermoDox: lyso-thermosensitive liposomal doxorubicin).

Secondly, nanocarriers encompass a wide range of materials, and not all of them have been tested extensively in humans. Hence, determining biocompatibility may be one of the major challenges. The majority of our toxicity studies are meant for materials used in bulk. However, nanoparticles have vastly different properties than the corresponding bulk matter; hence, standard toxicological profiling might not be sufficient to gauge or evaluate their toxicity. Even though preliminary results show that the nanocarriers are nontoxic and biodegradable, concerns regarding the long-term effects of using nanocarriers are still unknown. Moreover, nanoparticles accumulate in higher concentrations in specific organs, such as the liver, spleen, and lungs. Therefore, long-term toxicity studies of nanocarriers are essential before performing the clinical trials of nanomedicines.

Thirdly, a rigorous assessment of sterility and endotoxin in nanoparticles and nano-formulations is significant because most nanoparticles are used for intravenously delivered cancer treatments. These elements are neglected in the early stages of development, which may lead to various difficulties in the preclinical evaluation process. Endotoxins may originate from materials employed in the creation of nanoparticles, and sterility problems may result from machinery or equipment utilized during the formulation process.

Fourthly, nanocarriers can be synthesized using numerous methods depending on the type of nanomaterials as well as desired characteristics of nanocarriers. However, the most commonly used laboratory techniques include nanoprecipitation, ionic gelation, sonication, supercritical fluid technology, high-pressure homogenization, emulsion solvent evaporation, membrane extrusion, microfluidizer technology, coprecipitation, photolithography, etc. [182]. Many of these techniques, if not all, need close monitoring of various formulation parameters to achieve nanoformulation with desired reproducibility, since the characteristics of nanocarriers could be varied significantly with the subtle changes in these parameters. Supercritical fluid technology, high-pressure homogenization, membrane extrusion, and microfluidizer technology have satisfactory scale-up abilities among these techniques. Nevertheless, applications of these techniques to formulate multifunctional (e.g., surface-functionalized or TME-responsive) nanocarriers at a large scale are still doubtful. Furthermore, more improvement is required regarding drug loading capabilities and reproducibility. 

Last but not least, the clinical translation of nanomedicines is greatly hampered because experimental animal tumor models cannot accurately mimic the tumors in human clinical cancer. Their construction of experimental models that can be used to assess the pharmacokinetics and efficacy of nanomedicines must be carefully selected for the close resemblance of human tumors. 

Thus, the potential benefits of nanomedicines must be evaluated against concerns, including production costs, safety, and the complexity of nanoformulations. Clinical translation for some nanomedicines may never be achieved due to their associated cost and production needs, even though they outperform conventional formulations regarding therapeutic benefits. Depending on formulation and complexity, manufacturing costs for nanomedicine can be significantly higher than those for traditional pharmaceuticals. The environmental impact of commercial production is becoming increasingly relevant, considering not just the nanomaterials themselves, but also industrial waste and energy expenses. 

## 5. Conclusions

Cancer nanomedicine aims to address the inherent limitations of traditional cancer chemotherapy. The increased interest in using nanotechnology for cancer is mainly attributable to its potential to improve existing therapy with enhanced targeting capabilities, increase localized drug efficacy, minimize systemic toxicity, modulate drug release profiles, improve diagnostic sensitivity, strengthen imaging, and refine radiation therapy [183,184,185]. Additionally, nanocarriers can effectively transport a wide range of novel anticancer agents, such as nucleic acids, molecularly targeted drugs, and immunotherapeutics.

The development of theranostic nanomedicine offers a promising strategy for efficiently tracking the pharmacokinetics and accumulation of therapeutics, as well as disease progression. Similarly, numerous in vivo studies have shown that co-delivering multiple therapeutic agents using a single nanoformulation effectively circumvents drug resistance mechanisms, thereby showing enhanced tumor inhibition. Recently, nanotechnology has become more popular in the area of cancer immunotherapy in addition to chemotherapeutic delivery. Taken together, the future of nanomedicine seems promising, with advanced technology enhancing treatments and diagnostics and machine learning applications boosting to save substantial time and costs. Through an interdisciplinary approach, nanocarrier-based treatment can be envisioned in the foreseeable future. However, a detailed study of nanocarriers’ effectiveness and toxicity in suitable animal models must be performed to ensure a higher rate of clinical translation. 

## Figures and Tables

**Table 1:** Surface-functionalized lipid-based nanocarriers for active tumor targeting.

Target	Nanomaterial Description	Payloads	Cancer Type	Model	Reference
CD44 receptor	Anti-CD44 monoclonal antibody conjugated PEGylated liposomes; size: 107 ± 3.1 nm; ZP: −15.6 ± 0.3 mV	Doxorubicin	Colon carcinoma	In vitro: C-26 mouse colon adenocarcinoma cells In vivo: BALB/c mice bearing C-26 tumor	[54]
	Hyaluronic acid-coated SLNs; size: 224 ± 16 nm; ZP: −17.1 ± 0.73 mV	Docetaxel	Breast cancer	In vitro: MCF-7, MCF-7/ADR, and MDA-MBA-231 triple-negative human breast cancer cells	[55]
Epidermal growth factor receptor (EGFR)	EGFR-antagonistic affibody (Z_EGFR_)-conjugated PEGylated liposomes; size: 140.01 ± 0.84 nm; ZP: −13.40 ± 0.8 mV	Cisplatin	Epidermoid carcinoma	In vitro: A431 human squamous carcinoma cells In vivo: BALB/c nude mice bearing A431 tumor grafts	[56]
Estrogen receptor	Estrone-conjugated PEGylated liposomes; size: 129.53 ± 1.19 nm; ZP: −5.74 ± 0.51 mV	Epirubicin and paclitaxel	Breast cancer	In vitro: MCF-7 cells In vivo: MCF-7 tumor-bearing BALB/c nude mice	[57]
Folate receptor	Folic acid conjugated liposomes; size: 174.0 ± 0.9 nm; ZP: −8.5 mV	Celastrol and irinotecan	Breast and lung cancers	In vitro: MCF-7, MDA-MB-231, and A549 cells In vivo: MDA-MB-231 xenograft tumor-bearing BALB/c nude mice	[58]
	Folic acid conjugated NLCs; * NLC_(Gel-DOX-PEG-FA)_ size: 220 ± 46 nm and ZP: −24.5 ± 1.7 mV * NLC_(Pal-DOX-PEG-FA)_ size: 281 ± 18 nm and ZP: −28.0 ± 0.9 mV	Doxorubicin	Breast cancer	In vitro: MDA-MB-231 cells	[59]
Her2 receptor	MM-302 conjugated PEGylated liposomes; size: ~100 nm	Doxorubicin	Breast cancer	In vivo: HER2 expressing murine and human breast cancer mice models	[60]
p32 protein	LinTT1 peptide-functionalized liposomes; size: 146 ± 4 nm; ZP: −32.6 ± 2.3 mV	Doxorubicin and sorafenib	Breast cancer	In vitro: MCF-7 and MDA-MB-231 cells, MDA-MB-231 spheroids	[61]
Prostate-specific membrane antigen (PSMA)	Glutamate-Urea-Lysine conjugated PEGylated NLCs; size: 129 ± 3 nm; ZP: −36.3 ± 0.3 mV	Cabazitaxel	Prostate cancer	In vitro: LNCaP human prostate cancer cells	[62]
Transferrin receptor	Transferrin conjugated SLNs; size: 231.4 ± 2.5 nm; ZP: −8.36 ± 0.1 mV	Curcumin	Prostate cancer	In vitro: LNCaP cells In vivo: BALB/c nude mice bearing LNCaP tumors	[63]

* NLC(Gel-DOX-PEG-FA): Doxorubicin-loaded folic acid conjugated PEGylated nanostructured lipid carriers comprising Gelucire^®^43/01 solid lipid. NLC(Pal-DOX-PEG-FA): Doxorubicin-loaded folic acid conjugated PEGylated nanostructured lipid carriers comprising cetyl palmitate solid lipid.

**Table 2:** Nanotechnology-based approved cancer therapies.

Product	Nanocarrier	Drug	Indication	Manufacturer	Initial Approval Year
Doxil (Caelyx)	PEGylated liposome	Doxorubicin	Kaposi’s sarcoma, breast cancer, ovarian cancer, multiple myeloma	Janssen	FDA (1995) EMA (1996)
DaunoXome	Liposome	Daunorubicin	Kaposi’s sarcoma	Galen	FDA (1996)
Lipo-Dox	PEGylated liposome	Doxorubicin	Kaposi’s sarcoma, breast cancer, ovarian cancer	Taiwan Liposome	Taiwan (1998)
DepoCyt	Liposome	Cytarabine	Lymphomatous meningitis	Pacira Pharmaceuticals	FDA (1999)
Myocet	Liposome	Doxorubicin	Metastatic breast cancer	Teva UK	EMA (2000)
Abraxane	Albumin nanoparticle	Paclitaxel	Advanced NSCLC, metastatic breast cancer, metastatic pancreatic cancer	Abraxis BioScience/Celgene	FDA (2005) EMA (2008)
Oncaspar	Polymer protein conjugate	L-asparaginase	Acute lymphoblastic leukemia	Enzon-Sigma-Tau	FDA (2006)
Lipusu	Liposome	Paclitaxel	NSCLC, ovarian cancer, and breast cancer	Luye Pharma	State Food and Drug Administration of China (2006)
Genexol-PM	PEG-b-PLA polymeric micelle	Paclitaxel	Breast cancer, ovarian cancer, and NSCLC	Samyang Biopharmaceuticals	South Korea (2007)
Mepact	Liposome	Mifamurtide	Osteosarcoma	Takeda	EMA (2009)
NanoTherm	Iron oxide nanoparticle		Thermal ablation of glioblastoma, prostate cancer	MagForce Nano	EMA (2010) FDA (2018)
Onivyde	PEGylated liposome	Irinotecan	Metastatic pancreatic cancer	Merrimack Pharmaceuticals	FDA (2015)
DHP107	Lipid nanoparticle	Paclitaxel	Gastric cancer	Daehwa Pharmaceutical	South Korea (2016)
Vyxeos	Liposome	Daunorubicin: cytarabine (1:5 molar ratio)	Acute myeloid leukemia	Jazz Pharmaceuticals	FDA (2017) EMA (2018)
Apealea	Micelle	Paclitaxel	Ovarian, peritoneal, and fallopian tube cancer	Oasmia Pharmaceutical	EMA (2018)
Hensify	Hafnium oxide nanoparticle		Locally-advanced soft tissue sarcoma	Nanobiotix	CE mark (2019)

CE mark: European market approval; EMA: European Medicines Agency; FDA: U.S. Food and Drug Administration; NSCLC: non-small cell lung cancer.

**Table 3:** Nanotechnology-based formulations under different phases of clinical trials.

Product (Development Phase)	Sponsor	Active Ingredient	Nanoplatform	Indication	Status	Clinical Trial Number
Docetaxel-PNP (Phase 1)	Samyang Biopharmaceuticals Corporation	Docetaxel	Polymeric nanoparticles	Advanced solid malignancies	Completed	NCT01103791
ABT-888 (Phase 2)	AbbVie (prior sponsor, Abbott)	Temozolomide and lipo somal and doxorubicin	PEGylated liposomes	Ovarian cancer	Completed	NCT01113957
BIND-014 (Phase 2)	BIND Therapeutics	Docetaxel	Polymeric micelles	Second-line therapy for KRAS-positive or squamous cell NSCLC patients	Completed	NCT02283320
CPX-351 (Phase 2)	M.D. Anderson Cancer Center	Cytarabine and daunorubicin at 5:1 ratio	Liposomes	Acute myeloid leukemia	Completed	NCT02286726
LipoVNB (Phase 1/2)	Taiwan Liposome Company	Vinorelbine tartrate	Liposomes	Advanced malignancy	Completed	NCT02925000
NU-0129 (Early Phase 1)	Northwestern University	Small interfering RNAs (siRNAs) targeting the Bcl-2-like protein 12 (BCL2L12) sequence	Gold nanoparticles	Recurrent glioblastoma multiforme (GBM) or gliosarcoma	Completed	NCT03020017
iExosomes (Phase 1)	M.D. Anderson Cancer Center	KRAS G12D siRNA	Exosomes	Metastatic pancreas cancer with KrasG12D mutation	Recruiting	NCT03608631
CPC634 (CriPec^®^) (Phase 2)	Cristal Therapeutics	Docetaxel	Polymeric micelles	Ovarian cancer	Completed	NCT03742713
Cetuximab nanoparticles (Phase 1)	Ahmed A. H. Abdellatif	Cetuximab	Ethylcellulose nanoparticles	Colon cancer	Recruiting	NCT03774680
FF-10850 (Phase 1)	Fujifilm Pharmaceuticals	Topotecan	Liposomes	Advanced solid tumors	Recruiting	NCT04047251
Quantum dots coated with veldoreotide (Phase 1)	Al-Azhar University	Veldoreotide	Quantum dots	Breast cancer, skin cancer	Recruiting	NCT04138342
LY01610 (Phase 2)	Luye Pharma Group Ltd.	Irinotecan hydrochloride	Liposomes	Small cell lung cancer	Unknown	NCT04381910
INT-1B3 (Phase 1)	InteRNA	microRNA (miR-193a-3p)	Lipid nano-particles	Advanced solid tumors	Recruiting	NCT04675996
PRECIOUS-01 (Phase 1)	Radboud University Medical Center	Tumor antigen NY-ESO-1 and the iNKT cell activator threitolceramide-6 (ThrCer6, IMM60)	PLGA nanoparticles	Advanced solid tumor	Recruiting	NCT04751786
Mitoxantrone hydrochloride liposome injection (Phase 2)	CSPC ZhongQi Pharmaceutical Technology Co., Ltd.	Mitoxantrone hydrochloride	Liposomes	Breast cancer	Recruiting	NCT04927481
Liposomal bupivacaine (Phase 4)	Samaritan Health Services	Bupivacaine hydrochloride	Liposomes	Benign neoplasm	Recruiting	NCT05082441
WGI-0301 (Phase 1)	Zhejiang Haichang Biotech Co., Ltd.	AKT-1 antisense oligonucleotide	Lipid nanoparticles	Advanced solid tumors	Recruiting	NCT05267899
MagTrace (Phase 1/2)	Sahlgrenska University Hospital	Superparamagnetic iron oxide	Iron oxide nanoparticles	Deiagnostic test: Sentinel lymph node detection in breast cancer	Recruiting	NCT05359783
CDK-004 (Phase 1)	Codiak BioSciences	Antisense oligonucleotide targeting STAT6	Exosomes	Advanced hepatocellular carcinoma, gastric cancer metastatic to liver	Recruiting	NCT05375604
Nano-QUT (Phase 2)	Cairo University	Quercetin	PLGA-PEG nanoparticles	Oral cancer	Not yet recruiting	NCT05456022
OTX-2002 (Phase 1/2)	Omega Therapeutics	Biscistronic mRNA downregulate c-Myc expression	Lipid nanoparticles	Hepatocellular carcinoma	Recruiting	NCT05497453
Liposome doxorubicin (Phase 3)	Sun Yat-sen University	Doxorubicin	Liposomes	Desmoid tumor	Recruiting	NCT05561036
